# Distinction of surgically resected gastrointestinal stromal tumor by near-infrared hyperspectral imaging

**DOI:** 10.1038/s41598-020-79021-7

**Published:** 2020-12-14

**Authors:** Daiki Sato, Toshihiro Takamatsu, Masakazu Umezawa, Yuichi Kitagawa, Kosuke Maeda, Naoki Hosokawa, Kyohei Okubo, Masao Kamimura, Tomohiro Kadota, Tetsuo Akimoto, Takahiro Kinoshita, Tomonori Yano, Takeshi Kuwata, Hiroaki Ikematsu, Hiroshi Takemura, Hideo Yokota, Kohei Soga

**Affiliations:** 1 Department of Gastroenterology and Endoscopy, National Cancer Center Hospital East, Kashiwa, Chiba Japan; 2 Exploratory Oncology Research & Clinical Trial Center, National Cancer Center, Kashiwa, Chiba Japan; 3 Research Institute for Biomedical Sciences, Tokyo University of Science, Noda, Chiba Japan; 4 Department of Materials Science and Technology, Tokyo University of Science, Katsushika-ku, Tokyo, Japan; 5 Department of Mechanical Engineering, Tokyo University of Science, Noda, Chiba Japan; 6 Imaging Frontier Center, Tokyo University of Science, Noda, Chiba Japan; 7 Division of Radiation Oncology, National Cancer Center Hospital East, Kashiwa, Chiba Japan; 8 Course of Advanced Clinical Research of Cancer, Juntendo University Graduate School of Medicine, Bunkyo-ku, Tokyo, Japan; 9 Department of Gastric Surgery, National Cancer Center Hospital East, Kashiwa, Chiba Japan; 10 Department of Pathology and Clinical Laboratories, National Cancer Center Hospital East, Kashiwa, Chiba Japan; 11 RIKEN Center for Advanced Photonics, Wako, Saitama Japan

**Keywords:** Gastrointestinal cancer, Near-infrared spectroscopy, Cancer imaging, Cancer imaging

## Abstract

The diagnosis of gastrointestinal stromal tumor (GIST) using conventional endoscopy is difficult because submucosal tumor (SMT) lesions like GIST are covered by a mucosal layer. Near-infrared hyperspectral imaging (NIR-HSI) can obtain optical information from deep inside tissues. However, far less progress has been made in the development of techniques for distinguishing deep lesions like GIST. This study aimed to investigate whether NIR-HSI is suitable for distinguishing deep SMT lesions. In this study, 12 gastric GIST lesions were surgically resected and imaged with an NIR hyperspectral camera from the aspect of the mucosal surface. Thus, the images were obtained ex-vivo. The site of the GIST was defined by a pathologist using the NIR image to prepare training data for normal and GIST regions. A machine learning algorithm, support vector machine, was then used to predict normal and GIST regions. Results were displayed using color-coded regions. Although 7 specimens had a mucosal layer (thickness 0.4–2.5 mm) covering the GIST lesion, NIR-HSI analysis by machine learning showed normal and GIST regions as color-coded areas. The specificity, sensitivity, and accuracy of the results were 73.0%, 91.3%, and 86.1%, respectively. The study suggests that NIR-HSI analysis may potentially help distinguish deep lesions.

## Introduction

Gastrointestinal stromal tumor (GIST) is a submucosal tumor (SMT) originating from the digestive tract, and its predominant sites are the stomach (60%) and the small bowel (30%)^1^. Although some cases of GIST are discovered after initial symptoms such as pain, gastrointestinal (GI) bleeding, and bowel obstruction, most cases are asymptomatic and are revealed by GI examinations, including endoscopy.

In cases of gastric GIST, endoscopic examination is the primary tool for detection; the lesion first appears as an SMT. Clearly, this direct observation cannot differentially diagnose the SMT, because the lesions are mostly submucosal. Biopsies may have a low diagnostic yield, because the lesions are often deep and access is difficult^2^. Although endoscopic ultrasound-guided fine needle aspiration is a useful method for biopsy^3,4^, it can be technically demanding. Moreover, the definitive diagnosis of GIST requires time-consuming immunohistochemical procedures^5^. Thus, it would be desirable to develop a high-throughput simple diagnostic technique to identify GIST located under the mucosa. To this end, we suggest that near-infrared hyperspectral imaging (NIR-HSI) has the potential to become a key technology for diagnosing SMTs that are present deep within organs.

The NIR spectrum, ranging in wavelength from 800 to 2500 nm (wavenumber range: 12,500–4000 cm^−1^), has properties that makes it especially useful for bioimaging^6^. NIR light is less scattered by biological tissues than ultraviolet or visible light, and radiation absorption by water is much lower in the NIR spectrum than in the mid-infrared spectrum^7^. This tends to make tissues transparent to NIR wavelengths (~ 1 cm)^8^. Thus, the high transparency in the NIR spectrum makes non-destructive, non-invasive spectroscopic investigation of plants^9^ and human subjects possible^10–16^. Furthermore, NIR radiation excites biomolecules with an absorption level 100 times weaker than the wavelengths in the visible or mid-infrared spectrum. This allows safe and direct investigation of biomolecules in vivo. HSI is a potent imaging modality, that provides spectroscopic information with high spatial resolution (precision of measurement)^10–16^ and has been applied to various research fields, including distinction of epithelial tumors such as gastric cancer, without the use of fluorescent probes^17^. HSI using a machine-learning algorithm not only allows the acquirement of spectral information in each pixel of image data, but also the extraction of critical imaging data from large amounts of hyperspectral images^18,19^. However, very little progress has been made in the development of techniques for distinguishing deep lesions like GIST.

In this study, we aimed to investigate whether as a novel, minimally invasive diagnostic technique using GIST specimens, the NIR-HSI is suitable for the distinction of deep lesions.

## Results

**Figure 1:**
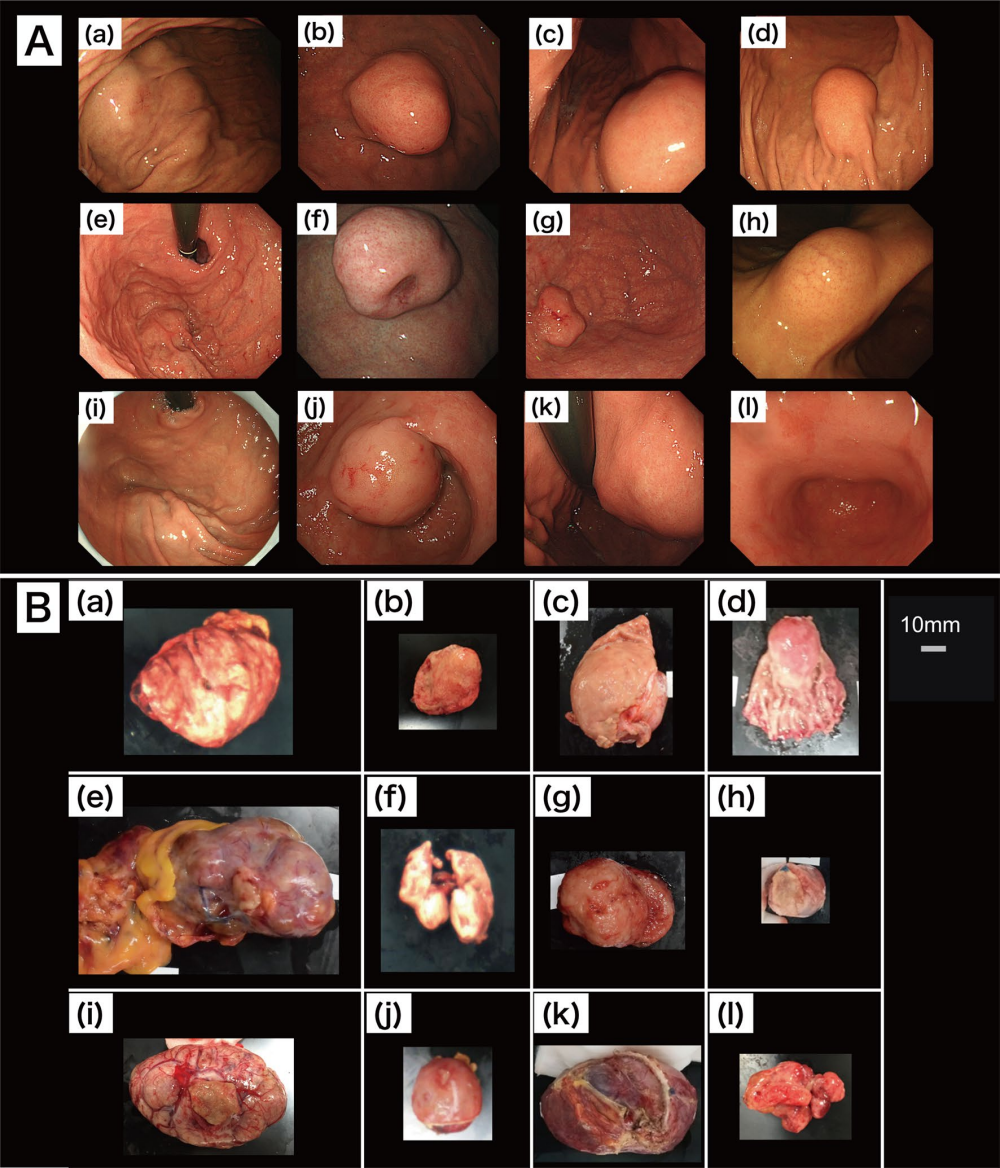
Endoscopic view and photographs of excised specimens of GIST. (**A**) (a–k) GIST can be seen as SMT protruding into the gastric lumen. (**A**) (l) SMT cannot be seen with an endoscope. (**B**) (a–l) Visible light photographs of specimens captured by a digital camera. *GIST* gastrointestinal tumor, *SMT* submucosal tumor.

**Figure 2:**
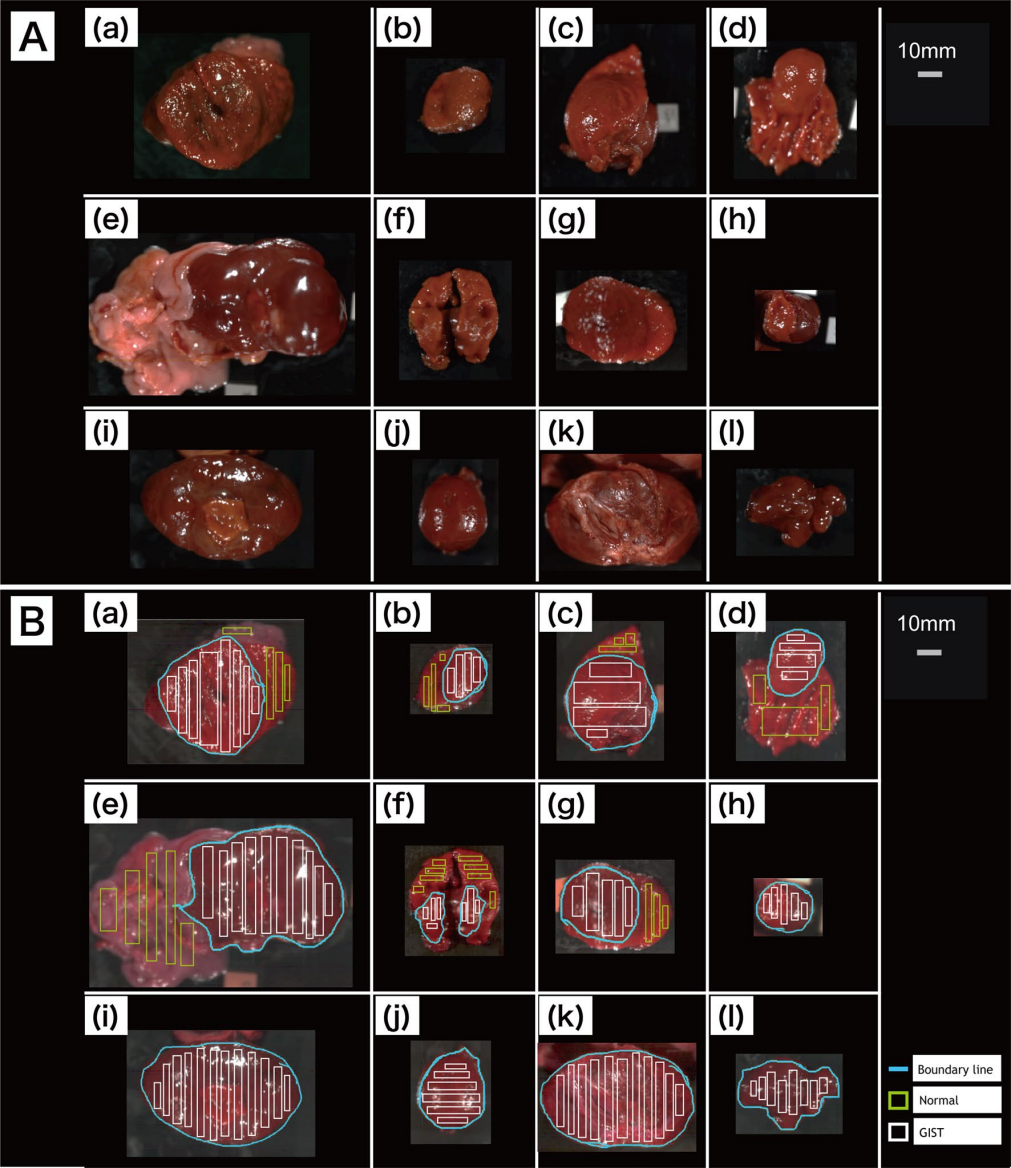
NIR image and preparation of training data. (**A**) (a–l) Pseudo-colored pictures of specimens captured by Compovision (NIR camera) (R: 1065 nm, G: 1280 nm, B: 1981 nm). (**B**) (a–l) Boundary line between GIST and normal region drawn by pathologist (blue), and bounding boxes for training data (green: normal tissue, white: GIST). *NIR* near-infrared, *GIST* gastrointestinal tumor.

Fourteen patients were screened, among whom 12 patients (10 men and 2 women) were enrolled and 2 were excluded (NIR-HSI images were acquired from the serosa side only). Their median age was 68 years (range 41–81 years). The median tumor size was 41 mm (range 24–80 mm). Endoscopic images of the lesions and pictures of excised 12 GIST specimens are shown in Fig. 1A,B. Figure 2A show pictures of 12 GIST specimens, captured by NIR-HSI. It can be seen that 7 specimens (a–d,f,g,j in Fig. 2A) were completely covered with mucosa, and 3 specimens (h,I,k in Fig. 2A) were partially covered with mucosa. In Fig. 2B, the boundary line of each lesion and bounding boxes have been drawn as described. Specimens presented in Fig. 2B(a–d) had both, GIST and normal tissue, including mucosa. Specimens shown in Fig. 2B(e–g) had GIST and other normal tissue, such as adipose tissue and submucosa, while all specimens presented in Fig. 2B(h–l) were GISTs.

**Figure 3:**
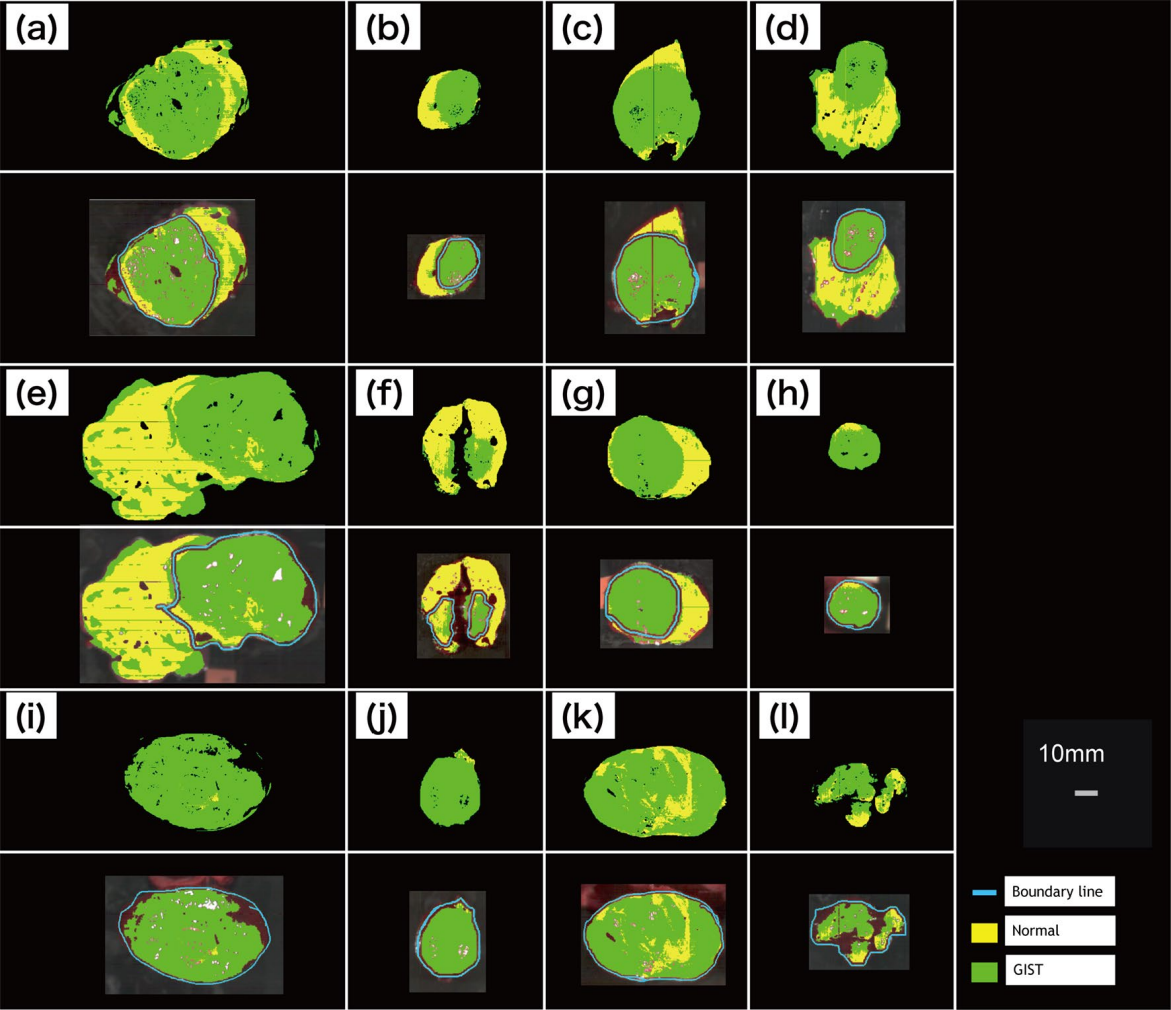
GIST region prediction analyzed by machine learning. ((**a**–**l**) upper images) The whole specimen. ((**a**–**l**) lower images) Merging of color-coded pixels and NIR picture with boundary line drawn by pathologist. The color-coded pixels near the boundary line were excluded. *GIST* gastrointestinal tumor, *NIR* near-infrared.

On the basis of training data, analysis of the spectra in HSI images was performed using the SVM algorithm, and GIST and normal regions were identified. The pixels that predicted GIST were colored green, while normal tissues were colored yellow. In Fig. 3, the upper images in (a–l) show the color-coded pixels predicted as GIST and normal tissue. The lower images in (a–l) were merged to include the pathologist’s boundary line, and the pixel areas used for the prediction-accuracy calculation can be seen. In the analyzed results, total pixels (371,053 px) were classified as follows: TP: 242,052 px, FN: 23,062 px, FP: 28,579 px, and TN: 77,360 px. From the classified pixels, the specificity, sensitivity, and accuracy were calculated as 73.0%, 91.3%, and 86.1%, respectively. The full results are listed in Table 1. The specimens in (h,l) were excluded from calculation of FPR and specificity, because they have only GIST regions.

**Table 1:** Prediction results of NIR-HSI analysis for the submucosal GIST region.

No.	Specimen size (W × D × H)	Total pixel number	FPR (%)	FNR (%)	Specificity (%)	Sensitivity (%)	Accuracy (%)
(a)	73 × 55 × 48	54,909	46.3	9.1	53.7	90.9	79.7
(b)	40 × 36 × 19	12,441	25.0	0.1	75.0	99.9	87.8
(c)	41 × 40 × 29	29,200	19.1	3.7	80.9	96.3	94.0
(d)	26 × 20 × 20	27,933	31.3	1.1	68.7	98.9	78.1
(e)	68 × 44 × 40	78,350	25.5	9.0	74.5	91.0	82.9
(f)	24 × 19 × 18	20,794	9.5	40.2	90.5	59.8	81.9
(g)	30 × 25 × 25	17,095	20.9	1.0	79.1	99.0	91.6
(h)	31 × 30 × 21	7930	–	6.7	–	93.3	93.3
(i)	77 × 54 × 48	49,779	–	0.8	–	99.2	99.2
(j)	32 × 28 × 25	10,544	–	1.1	–	98.9	98.9
(k)	80 × 52 × 48	51,885	–	17.0	–	83.0	83.0
(l)	44 × 28 × 25	10,193	–	25.6	–	74.4	74.4

*NIR* near-infrared, *HSI* hyperspectral imaging, *GIST* gastrointestinal tumor, *W* width, *D* diameter, *H* height, *FPR* false-positive rate, *FNR* false-negative rate.

**Figure 4:**
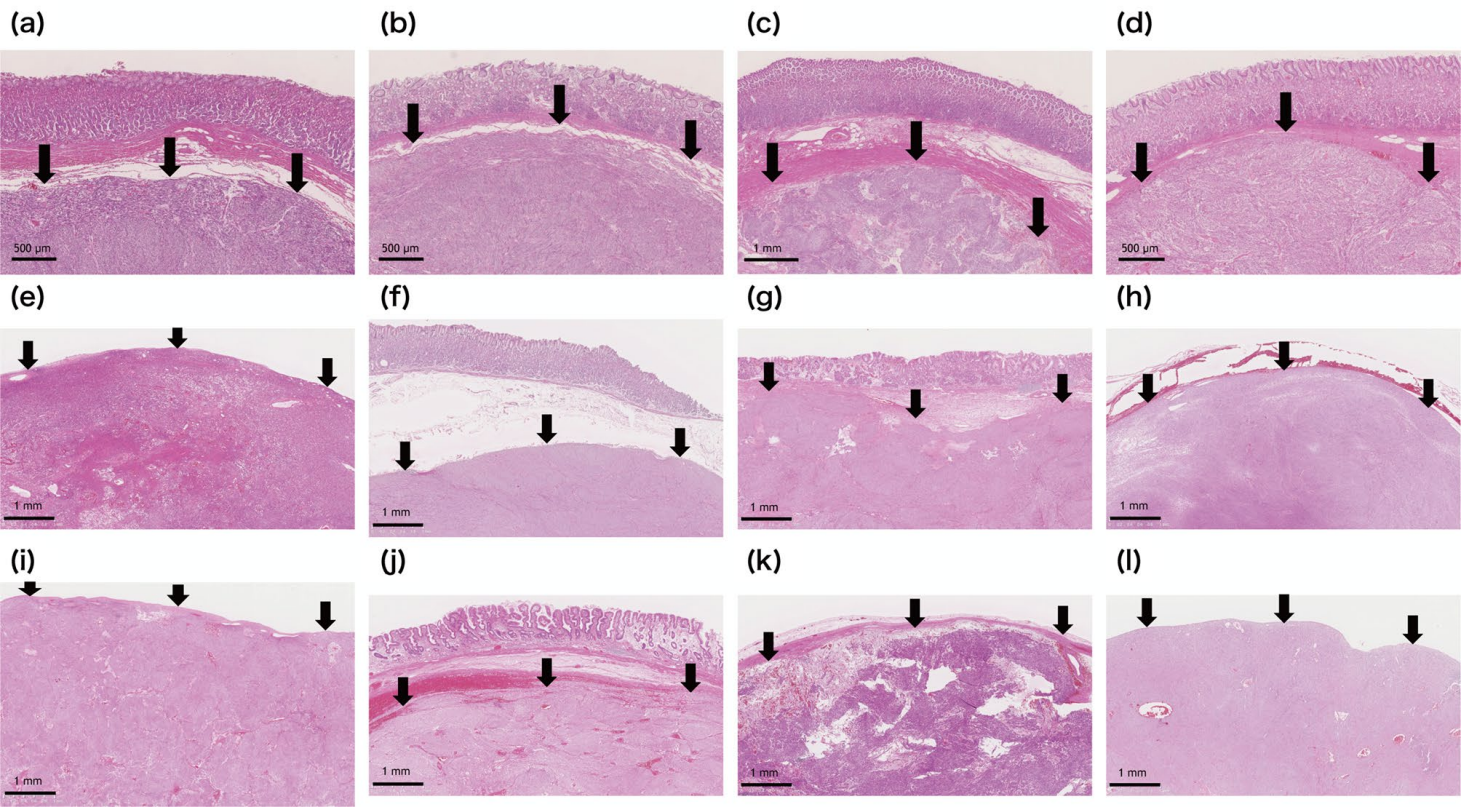
Histopathological observations of GIST by H&E staining (×5). (**a**–**d**,**f**,**g**,**j**) The tumor (black arrows) located in the submucosal layer. (**e**,**h**,**i**,**k**,**l**) the tumor not covered by normal mucosa. *GIST* gastrointestinal tumor.

Figure 4 shows microscopic images of GIST specimens stained by H&E staining. The black arrows indicate the upper border of GIST lesions. The depth from the surface of the mucosa to the upper border of the lesions was 0.4–2.5 mm (a–d,f,g,j). The diagnosis of GIST was confirmed by immunohistochemical analysis. Eleven specimens (a–k) were c-kit positive and 1 specimen (l) was c-kit and CD-34 negative, but DOG-1 positive.

## Discussion

To the best of our knowledge, this is the first report demonstrating the distinction of SMT using NIR-HSI. In this study, GIST lesions were predicted with 86.1% accuracy in 12 specimens using the machine-learning algorithm. The number of GIST specimens in this study was relatively small, because it is an extremely rare disease with an incidence of 1–2 in 100,000 people per year^20^. However, in this study, NIR-HSI captured pixels (total pixel: 371,053 px) were classified using the leave-one-out cross-validation procedure. Therefore, the sample size is considered to be sufficient, and its generalizability has been demonstrated. In addition, GIST is thought to arise from clonal expansion of transformed interstitial cells of Cajal in the stomach wall^21^, and are expected to show little variation in terms of histology. Therefore, we believe that the number of GIST specimens in this study was adequate for distinguishing GIST from normal mucosa.

**Figure 5:**
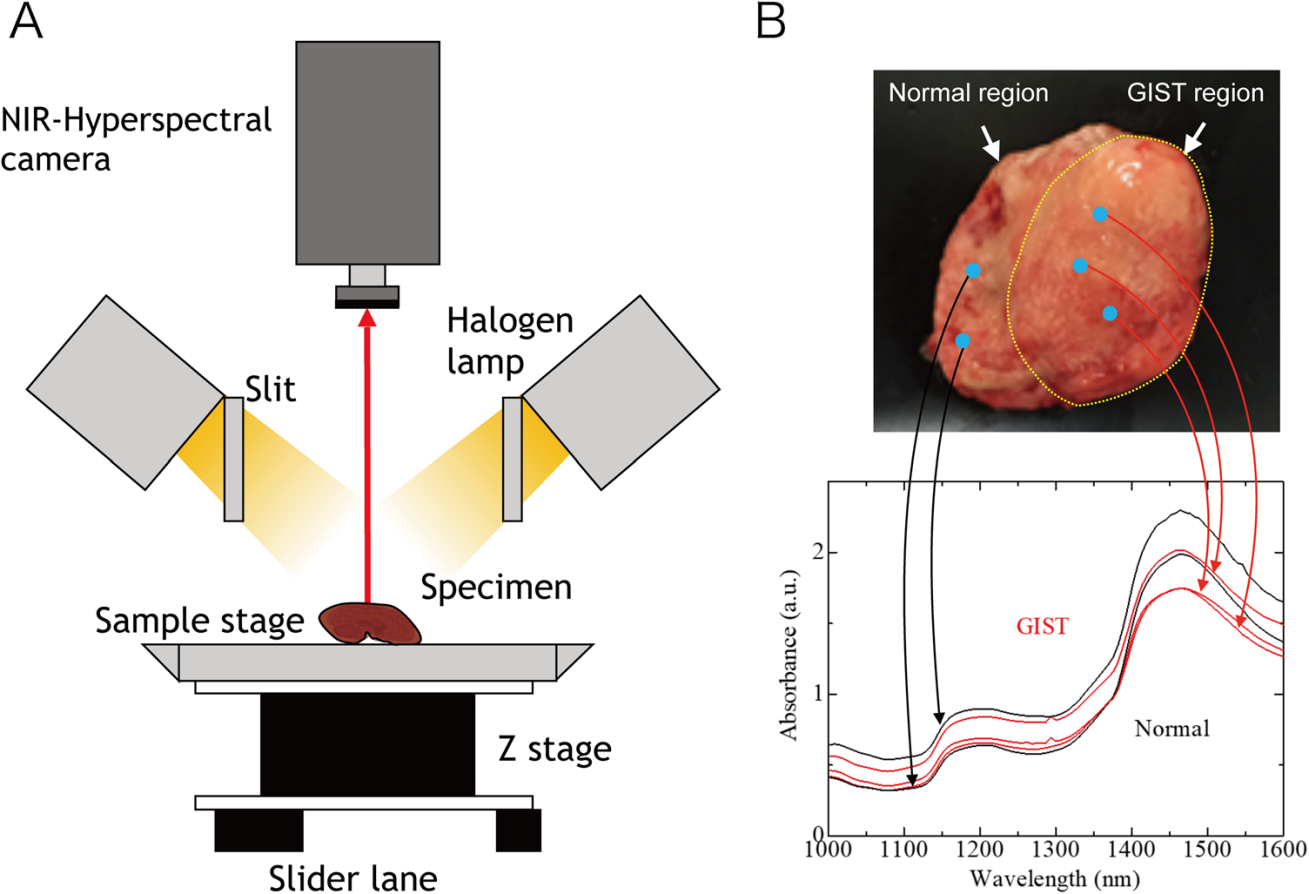
NIR-HSI system and absorbance spectra. (**A**) Setup of NIR-HSI system. (**B**) NIR absorbance spectra of HSI pixels of the GIST (Red) and normal (Black) regions. *NIR* near-infrared, *HSI* hyperspectral imaging, *GIST* gastrointestinal tumor.

Considering the results in the view of distinction of deep lesion, 10 of 12 specimens were completely or partially covered with mucosa, and the submucosal GIST lesions were identified using our method. This suggests that some GIST specific spectra information can be distinguished by NIR transparency. However, from the spectra (shown in Fig. 5B), we can infer that the difference between an NIR image of a GIST lesion and that of normal tissue may be difficult to distinguish from raw data of the NIR-HSI scan, as the spectra of GIST and normal tissue are similar. Therefore, to predict the site of the GIST lesion, machine-learning analysis with its datasets of GIST and normal reference images is essential.

The specificity and sensitivity of GIST distinction were 73.0% and 91.3%, respectively. In a previous study, Akbari et al*.* reported on the prediction of gastric cancer lesions on the mucosa, using SVM^17^. The results showed a specificity and sensitivity of 80% and 73%, respectively. Our results were similar, even though the lesions in our study were submucosal.

Although the border between GIST and normal tissue can be seen relatively clearly (Fig. 3), some analyzed specimens had high FNR or FPR. In the case of low-specificity specimens, such as those presented in Fig. 3a,d (53.7 and 68.7%, respectively), the green area outside the blue border line can be found largely at the edge of the specimens. This suggests that the NIR absorbance was poor, because the tissue at the edge was thin. However, there will be no edge in vivo; therefore, the impact of this effect can be reduced. In the case of the low sensitivity specimen (Fig. 4f [59.8%]), the lesion was covered with over 2 mm of mucosa and submucosa. We suggest that the NIR absorbance information of the lesion was reduced because of the thickness of the covering tissue. In addition, the lesion was the smallest (5824 px) of all the specimens; hence, we considered that even a small number of FN pixels may have affected the sensitivity. In any specimen, uneven surfaces can cause shadows and scattering of the NIR radiation, which lead to FN and FP errors. For example, the specimen presented in Fig. 2A(l) has an uneven surface and a relatively high FNR (25.6%). To diagnose smaller lesions and improve accuracy in the future, the following areas of improvement have been identified: creation of a much larger training dataset, addition of depth-of-lesion information to training data, and capture of high quality data by scanning lesions on as smooth a surface as possible.

Some SMT that need to be differentiated from GIST are myogenic tumors, such as leiomyoma and leiomyosarcoma, neurogenic tumors, including schwannoma and neurofibroma, and vascular tumors^22^. Owing to the limitations of conventional endoscopy, other techniques, such as endoscopic ultrasonography, contrast-enhanced computed tomography, or MRI, may be helpful in the diagnosis. High-risk features obtained by these techniques, such as irregular borders, homogeneous internal echoes including anechoic areas and heterogeneous enhancement, are known to be highly associated with GIST or malignant SMT. However, none of these features are specific for either condition^23^.

Development of an endoscopic NIR-HSI system is under way. It will enable clinicians to identify GISTs and differentiate them from other gastric SMTs. Moreover, it is anticipated that such a device can be applied to qualitative diagnosis of the extent and depth of epithelial tumors. Another area of interest for the future is the estimation of GIST malignancy. The genetic mutations in GIST are well correlated with imatinib activity^23^. For example, *KIT* exon 11 mutated GISTs are very sensitive to imatinib, while wild-type GIST (defined as GIST without any mutations in the *KIT* and *PDGFRA* genes) is insensitive to imatinib, and has a poor prognosis^23^. Obtaining such information before treatment could offer great benefits in selecting the best treatment and predicting the prognosis.

The main limitation of the study was that gastric SMTs other than GIST were not analyzed. The SMTs that we studied were limited to surgical specimens with a confirmed diagnosis of GIST. In clinical practice, it is important to be able to differentiate GISTs from other gastric SMTs by endoscopy; therefore, the analysis of other SMTs will be necessary. We have now developed an NIR-HSI device, that can be inserted into the instrument port of an endoscope (ø 3.2 mm), and have successfully performed NIR-HSI scans. We expect to perform endoscopic NIR-HSI analysis in the near future.

In conclusion, this study showed that, NIR-HSI analysis aided by machine-learning can identify differences between GIST and normal tissue with high prediction accuracy, specificity, and sensitivity. These results, indicate great potential for the clinical use of NIR-HSI in the diagnosis of SMT such as GIST.

## Methods

### Collection of surgical specimens and definitive diagnosis

Patients with clinically diagnosed GIST who underwent surgery between April 2016 and March 2018 were examined in this study. The inclusion criteria were: (i) clinical diagnosis of GIST; (ii) age of 20 years or older; and (iii) written informed consent for this study. The exclusion criteria were: (i) a history of prior chemotherapy; (ii) presence of hepatitis B virus surface antigen or hepatitis C virus antibody; and (iii) judged inappropriate for this study, such as improperly captured images. The indications for surgery were according to clinical practice guidelines for GIST in Japan^20^. This study was approved by the Institutional Review Board of the National Cancer Center Japan (approval no. 2015-339) and conforms to the provisions of the Declaration of Helsinki and the Epidemiological Study Guideline issued by the Japan Ministry of Health, Labor, and Welfare. All patients provided written informed consent before inclusion.

### Near-infrared hyperspectral image capture

An imaging system with a high-speed NIR hyperspectral camera (Compovision, CV-N800HS; Sumitomo Electric Industries, Ltd., Osaka, Japan) was used to obtain NIR-HSI images (wavelength: 1000–2350 nm; wavelength resolution: 6.3 nm). The detector (NIR spectroscopic camera), captured data values for each wavelength band, on each pixel per line of the image, in one scan. By scanning multiple lines (by sliding the sample stage), three-dimensional HSI images (*x*–*y*–λ axes) were obtained, producing a virtual “data cube” for processing and analysis. A schematic illustration and spectral data obtained are shown in Fig. 5A,B**.** An aluminum plate with a collimating slit was placed between the halogen lamp and the tissue sample to prevent damage to specimens by heat.

Each of the fresh specimens which are resected from stomach were placed on the sliding stage without trimming, and NIR-HSI images were acquired from the mucosal side under illumination from a halogen lamp (0.96 W/cm^2^). The resultant temperature rise was up to 3.3 °C. The integration time and frame rate for each wavelength band was set at 2.5 ms and 320 frames/s, respectively. Visible light images of the fresh specimens were also captured by a digital camera. The acquired raw images were calibrated using white and dark reference images. After capturing the NIR-HSI images, all surgical specimens were fixed with formalin, stained with H&E, and subjected to immunohistochemical examinations, including c-kit, CD34, and, if necessary, Discovered On GIST-1 (DOG1).

### Machine learning

In the machine learning part of the study, NIR-HSI images of all specimens were used as the dataset for analysis. Spectra of > 1600 nm wavelength were removed from the analysis because of lower sensitivity of the NIR camera and high absorption by water in those bands. In addition, in the 1300 nm spectra, reflectance rates of over 70% and below 10% were defined as highlights and shadows, respectively, and these pixels were removed from the dataset.

To create two regions showing pixels of normal mucosa and of the GIST lesion, a boundary line was drawn by a pathologist. The areas inside and outside were defined as “GIST” and “normal,” respectively. Bounding boxes were made, guaranteed to be GIST or normal tissue, and the spectra of each were used as training data for the algorithm.

Leave-one-out cross-validation was employed, because it is suggested that the procedure provides an almost unbiased estimate of the generalization ability^24^. In the leave-one-out cross-validation procedure, data sets are constructed excluding training data of a specimen for testing.

A machine learning algorithm, support vector machine (SVM), has often been found to provide higher classification accuracies than the maximum likelihood and the multilayer perceptron neural network classifiers in HSI classification^25^. Therefore, SVM was employed. This algorithm works by solving an optimization problem as follows^26^:

$$\mathrm{minimize} \,t\left({w}_{n},{\xi }_{i}\right)=\frac{1}{2}\sum_{n=1}^{k}{\parallel {w}_{n}\parallel }^{2}+\frac{C}{m}\sum_{i=1}^{m}{\xi }_{i}$$

1 $$\mathrm{subject\, to} \langle {x}_{i},{w}_{{y}_{i}}\rangle -\langle {x}_{i},{w}_{n}\rangle \ge {b}_{i}^{n}-{\xi }_{i} (i=1,\dots ,m)$$

2 $$\mathrm{where}\, {b}_{i}^{n}=1-{\delta }_{{y}_{i},n}$$

where the decision function is

3 $${argmax}_{m=1,\dots ,k} \langle {x}_{i},{w}_{n}\rangle $$

This optimization problem is solved by a decomposition method^27^. In this study, we used C = 1 and the RBF kernel as follows:

4 $$K\left({x}_{i},{x}_{j}\right)=\mathrm{exp}\left(-\frac{{\parallel {x}_{i}-{x}_{j}\parallel }^{2}}{{\sigma }^{2}}\right)$$

where the optimal values of the hyper-parameter of σ^2^ are estimated as follows:

5 $$ \sigma ^{2} { := }median~\left\{ {\parallel x_{i}  - x_{j} \parallel ^{2} ~\left| {i < j} \right|} \right\} $$

In NIR spectral measurement, it is reported that variance is caused, such as non-specific scatter at the surface of the sample^28^. To reduce the variance, the standard normal variate (SNV) was used for baseline correction of the spectrum as follows:

6 $$\mathrm{Z}=\frac{\mathrm{x}-\mathrm{mean}\left(\mathrm{x}\right)}{\mathrm{std}\left(\mathrm{x}\right)}$$

where x is a row vector containing the original spectrum, mean(x) is the mean of x, std(x) is the standard deviation of x, and z denotes the SNV-transformed spectrum.

### Calculation of prediction accuracy

To evaluate prediction accuracy, the coordinates of the prediction pixels were compared with boundary line images; the pixels were classified in to four groups as follows: GIST is predicted as GIST (true-positive: TP), GIST is predicted as normal (false-negative: FN), normal is predicted as GIST (false-positive: FP), normal is predicted as normal (true-negative: TN). From the classified pixels, the false-positive rate (FPR) and false-negative rate (FNR) can be calculated; the specificity, sensitivity, and accuracy were defined as follows:

7 $$Specificity \left[\%\right]=\frac{TN}{FP+TN}\times 100$$

8 $$Sensitivity \left[\%\right]=\frac{TP}{TP+FN}\times 100$$

9 $$Accuracy \left[\%\right]=\frac{TP+TN}{TP+TN+FP+FN}\times 100$$

For the accuracy calculation, the thickness of the boundary line was doubled both, on the inside and on the outside of the line, and the data found were excluded because the boundary line was drawn freehand by the pathologist, and was therefore susceptible to error.
