# Author Correction: Distinction of surgically resected gastrointestinal stromal tumor by near-infrared hyperspectral imaging

**DOI:** 10.1038/s41598-021-98867-z

**Published:** 2021-09-20

**Authors:** Daiki Sato, Toshihiro Takamatsu, Masakazu Umezawa, Yuichi Kitagawa, Kosuke Maeda, Naoki Hosokawa, Kyohei Okubo, Masao Kamimura, Tomohiro Kadota, Tetsuo Akimoto, Takahiro Kinoshita, Tomonori Yano, Takeshi Kuwata, Hiroaki Ikematsu, Hiroshi Takemura, Hideo Yokota, Kohei Soga

**Affiliations:** 1 Department of Gastroenterology and Endoscopy, National Cancer Center Hospital East, Kashiwa, Chiba Japan; 2 Exploratory Oncology Research & Clinical Trial Center, National Cancer Center, Kashiwa, Chiba Japan; 3 Research Institute for Biomedical Sciences, Tokyo University of Science, Noda, Chiba Japan; 4 Department of Materials Science and Technology, Tokyo University of Science, Katsushika‑ku, Tokyo, Japan; 5 Department of Mechanical Engineering, Tokyo University of Science, Noda, Chiba Japan; 6 Imaging Frontier Center, Tokyo University of Science, Noda, Chiba Japan; 7 Division of Radiation Oncology, National Cancer Center Hospital East, Kashiwa, Chiba Japan; 8 Course of Advanced Clinical Research of Cancer, Juntendo University Graduate School of Medicine, Bunkyo‑ku, Tokyo, Japan; 9 Department of Gastric Surgery, National Cancer Center Hospital East, Kashiwa, Chiba Japan; 10 Department of Pathology and Clinical Laboratories, National Cancer Center Hospital East, Kashiwa, Chiba Japan; 11 RIKEN Center for Advanced Photonics, Wako, Saitama Japan

Correction to: *Scientific Reports* 10.1038/s41598-020-79021-7, published online 14 December 2020

The original version of this Article omitted an affiliation for Daiki Sato. The correct affiliations are listed below.

Department of Gastroenterology and Endoscopy, National Cancer Center Hospital East, Kashiwa, Chiba, Japan

Course of Advanced Clinical Research of Cancer, Juntendo University Graduate School of Medicine, Bunkyo-ku, Tokyo, Japan

The original Article has been corrected.

